# Mathematical Estimation of Endogenous Proline as a Bioindicator to Regulate the Stress of Trivalent Chromium on Rice Plants Grown in Different Nitrogenous Conditions

**DOI:** 10.3390/toxics11100803

**Published:** 2023-09-22

**Authors:** Chengzhi Li, Yuxi Feng, Peng Tian, Xiaozhang Yu

**Affiliations:** College of Environmental Science & Engineering, Guilin University of Technology, Guilin 541004, Chinayu-xifeng@foxmail.com (Y.F.);

**Keywords:** proline, rice, trivalent chromium, nitrogen source, mass balance

## Abstract

The accumulation of proline impacts the defense mechanisms of plants against the harmful effects of adverse environmental conditions; however, its concentration in plants is associated with the metabolism of N. Therefore, the effects of exogenous organic [glutamate (Glu)/arginine (Arg)] and inorganic [nitrate (NO_3_^−^)/ammonium (NH_4_^+^)] N on the accumulation of proline (Pro) in rice plants under trivalent chromium [Cr(III)] stress were studied through using the mass balance matrix model (MBMM). Application of ‘NH_4_^+^’ showed the largest contribution to the Pro content in rice shoots under different concentrations of Cr(III), followed by ‘NO_3_^−^’, ‘Arg’, and ‘Glu’ applications. On the other hand, ‘Arg’ application displayed the largest contribution to the Pro content in roots under Cr(III) stress, followed by ‘NH_4_^+^’, ‘Glu’, and ‘NO_3_^−^’ applications. The combined application of ‘NH_4_^+^+Arg’ showed the greatest contribution to the Pro content in both roots and shoots of Cr(III)-treated rice seedlings, while the application of ‘NO_3_^−^+Glu’ showed the least contribution to the Pro content in rice seedlings. The current study indicated that the endogenous level of Pro in rice seedlings is quite sensitive to Cr(III) stress under different N sources, and the mathematical modeling showed a reliable result while estimating the relationship between Pro content and N source application.

## 1. Introduction

Chromium (Cr) is widely distributed in the environment through different activities of industries such as metallurgical and chemical [1]. Due to its high solubility, mobility, and oxidizing potential, Cr is considered as one of the top 20 hazardous materials to be remediated on an early basis [2]. Naturally, Cr can exist in several oxidation states, ranging from “−2” to “+6”, in which the hexavalent [Cr(VI)] and trivalent chromium [Cr(III)] are the most stable forms of Cr [1]. Continuous input of Cr into the environment from the industrial sector makes it a serious threat to plants [3,4]. Although plants uptake a small amount of Cr from the soil, its over-accumulation in plants affects their nitrogen (N) metabolism. For example, Cr exposure imbalanced the assimilation of nitrate (NO_3_^−^) and ammonium (NH_4_^+^) by modifying the activities of nitrate reductase (NR), nitrite reductase (NiR), glutamine (GS), and glutamate dehydrogenase (GDH) in *Sorghum bicolor* and *Solanum. lycopersicum* (Martins et al. [5]).

Nitrogen is a crucial macroelement for supporting plant growth and development [6], wherein NO_3_^−^ and NH_4_^+^ are the most available inorganic forms of N for plants. Uptake and subsequent assimilation of NO_3_^−^ or NH_4_^+^ by plants play a crucial role in improving plant growth and yield [7]. It provides the building blocks for the synthesis of a plethora of biomolecules, such as proteins, nucleic acids, and chlorophyll. Among the biomolecules, amino acids are the major component of plant biomass [8]. Proline (Pro), a common amino acid, plays an important role in plants. For instance, it maintains osmotic balance, protects subcellular structures, scavenges reactive oxygen species (ROS), stabilizes protein and DNA, and provides N sources in responses to stress conditions including drought, high temperature, salinity, UV radiation, pathogens, and chemical exposure [9]. In addition to acting as an excellent osmolyte, proline also plays a major role as a metal chelator, an antioxidative defense molecule, and a signaling molecule [10]. Therefore, the level of Pro accumulation in plant tissues has been suggested as a sensitive indicator to evaluate the overall performance of plant growth in various contaminated sites. It has been reported that the content of amino acids in plants is highly dependent on N nutrition [11]. Our previous study also confirmed that NH_4_^+^-fed rice seedlings showed a significantly dose-dependent increase in Pro in shoots, while the innate level of Pro in NO_3_^−^-fed rice seedlings is independent of the NO_3_^−^ dose supplied. Additionally, we noticed that accumulation of Pro was observable in rice plants supplied with additional Glu and Arg, where the latter demonstrated much higher potential than the former during the synthesis of Pro in rice plants [12].

Unfavorable environmental conditions inhibit plant growth and development by altering various biological processes including N metabolism. In this case, a change in the N cycle could alter the composition and the synthesis of amino acids [13]. Literature on the effect of chromium toxicity on enzymes of nitrogen metabolism is available [14]. It is also reported that fertilization of inorganic N (NO_3_^−^ and NH_4_^+^) can influence the bioavailability and toxicity of Cu, Cd, and Cr in plants by altering the synthesis of organic molecules (with N), e.g., Pro, Glu, and Arg [15,16]. Our previous study reported that the innate level of Pro in rice plants is changeable due to the application of different N sources (NO_3_^−^ and NH_4_^+^) under Cr stress [17]. Rice is one of the world’s most produced crops and a major energy source in the world. Research on the synthesis of Pro in rice plants from both inorganic (NO_3_^−^ and NH_4_^+^) and organic (Arg and Glu) N sources showed a completely different increment pattern [12]. However, no information is available to investigate the endogenous Pro in rice plants fertilized with different nitrogenous chemicals under Cr(III) stress. Therefore, we hypothesized that exogenous N affects the accumulation of proline (Pro) in rice plants under trivalent chromium.

To achieve the objective, we carried out the present study in the following manner: (1) determined the content of Pro in rice tissues under Cr(III) stress with different inorganic (NO_3_^−^ and NH_4_^+^) and organic (Arg and Glu) N sources alone and in combination; (2) mathematically evaluated the contribution of different N sources alone and in combination to Pro content in rice plants under Cr(III) stress, based on the mass balance matrix model (MBMM); (3) predicted a suitable combination of different N sources for regulation of Cr(III) stress in rice plants using the content of Pro as a bioindicator. Overall, our study provides a new method to elucidate the contribution of different N sources to Pro accumulation in rice plants under Cr(III) stress.

## 2. Methods and Materials

### 2.1. Rice Seedlings and Cr Treatment

**Figure 1:**
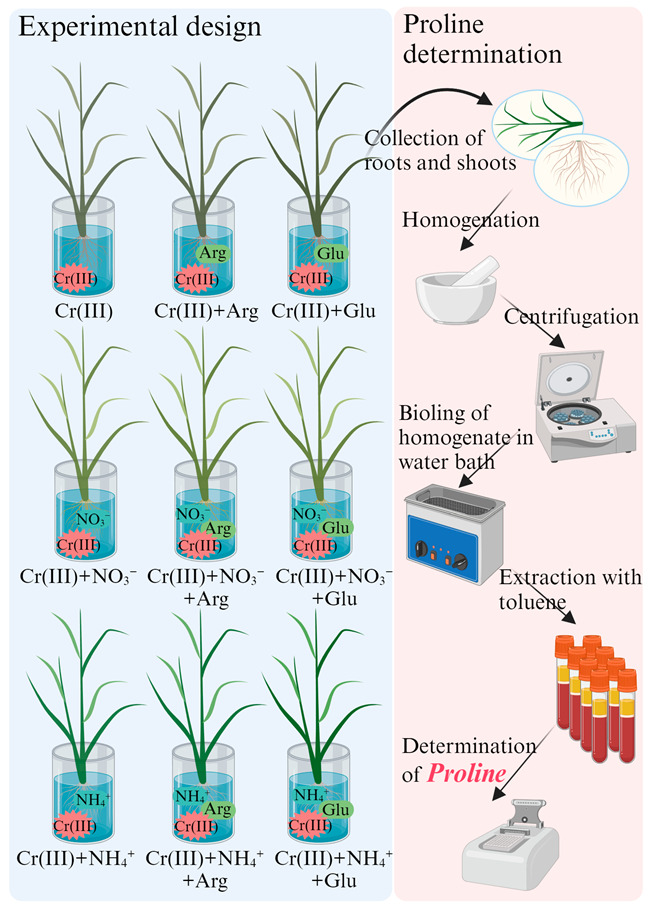
The design of experimental treatments.

Rice (*Oryza sativa* L. XZX 45) seedlings were obtained using the method described in our previous study [6]. Briefly, after soaking the seeds with deionized water for 24 h, the seeds were cultivated in sand soils and placed in a growth chamber (temperature: 25 ± 0.5 °C and relative humidity: 60 ± 2%). Rice seedlings were irrigated with a modified ISO8629 nutrient solution during the entire growth period, i.e., 16 days [1]. Seedlings of similar sizes were selected for the following treatments (Figure 1).

(1) ‘Cr(III)+(−N_I_)’ treatments: Rice seedlings were pre-treated with the nutrition solution without KNO_3_/NH_4_Cl (‘−N_I_’), but with 3 mM Arg (+N_Arg_) or 10 mM Glu (+N_Glu_) for 12 h [12], and subsequently exposed to Cr(III) solution at 0, 12.0, 24.0 and 40.0 mg Cr/L for 2 days (Figure 1).

(2) ‘Cr(III)+(+N_NO3_^−^)’ treatments: Rice seedlings were pre-treated with the KNO_3_-containing nutrient solution (+N_NO3_^−^) with 3 mM Arg (+N_Arg_) or 10 mM Glu (+N_Glu_) for 12 h, respectively [12], and subsequently exposed to Cr(III) solution at 0, 12.0, 24.0 and 40.0 mg Cr/L for 2 days (Figure 1).

(3) ‘Cr(III)+(+N_NH4_^+^)’ treatments: Rice seedlings were pre-treated with the NH_4_Cl-containing nutrition solution ‘+N_NH4_^+^’, with 3 mM Arg (+N_Arg_) or 10 mM Glu (+N_Glu_) for 12 h [12], and exposed to Cr(III) solution at 0, 12.0, 24.0 and 40.0 mg Cr/L for 2 days (Figure 1).

The symbol ‘−N_O_’ indicates N treatment without Arg or Glu; ‘+N_Arg_’ indicates N treatment with Arg; ‘+N_Glu_’ indicates N treatment with Glu; ‘−N_I_’ indicates the N treatment without KNO_3_ or NH_4_Cl; ‘+N_NO3_^−^’ indicates KNO_3_ treatment’; ‘+N_NH4_^+^’ indicates NH_4_Cl treatment. The weight of KNO_3_ and NH_4_Cl in the nutrient solution is equal to 39.5 mg N/L. All glass containers were wrapped with aluminum foil to minimize water loss and inhibit algae growth. Each treatment was prepared in four biological replicates. All chemicals used were of analytical grade and purchased from Aladdin Chemistry Co., Ltd. (Shanghai, China).

### 2.2. Measurement of Relative Growth Rate

Rice seedlings were weighed before and after exposure to Cr(III) treatments. The relative growth rate (RGR, %) was calculated based on our previous study [12].

### 2.3. Measurement of Pro Content in Rice Seedlings

After 2 days of exposure, rice seedlings were divided into roots and shoots. Subsequently, these tissues were homogenized in a pre-chilled mortar with 3% sulfosalicylic acid (5 mL). The homogenate was transferred to a 10 mL tube for centrifuging (4 °C, 11,000 rpm, 15 min). After centrifugation, 2 mL of the supernatant was taken and mixed with equal quantity, i.e., 2.0 mL of glacial acetic acid and 2.5% ninhydrin (glacial acetic acid: 6 mol/L phosphoric acid, 60:40) solution, and boiled in a water bath for 1 h. For instant cooling, the solution was kept on ice for 5 min followed by extraction of desired product using 4 mL toluene. The amount of Pro content was estimated using a spectrophotometer at 520 nm against a toluene reference, as per standard protocol formulated by Li et al. [12]. A standard curve was constructed to measure proline content between the absorbance at 520 nm and L-proline content (Supplementary Materials). Series content of L-proline was 0, 2, 4, 6, 8, and 10 μg/mL of ddH_2_O. A linear regression was observed between the absorbance values at 520 nm and L-proline contents at 0–10 μg (R^2^ = 0.9992).

### 2.4. Modeling the “Mass Balance Matrix”

In this study, we developed a “mass balance matrix” model (MBMM), based on the elementary rows (*r*)/columns (*c*) transformation to predict the optimal tolerance strategies for rice seedlings grown in different N sources under Cr(III) stress using the content of Pro as the dependent variable. Accordingly, the contribution of different N sources to Pro content was estimated. The elements of all matrixes are denoted by *a_ij_* (*i*, *j* = 1, 2, 3).

The fundamental matrix is as follows:

(1) $$\left[\begin{array}{ccc} \left(-N_{O})\&(-N_{I}\right) & \left(+N_{\mathrm{Arg}})\&(-N_{I}\right) & \left(+N_{\mathrm{Glu}})\&(-N_{I}\right)\\ \left(-N_{O})\&(+N_{{\mathrm{NO}}_{3}^{-}}\right) & \left(+N_{\mathrm{Arg}})\&(+N_{{\mathrm{NO}}_{3}^{-}}\right) & \left(+N_{\mathrm{Glu}})\&(+N_{{\mathrm{NO}}_{3}^{-}}\right)\\ \left(-N_{O})\&(+N_{{\mathrm{NH}}_{4}^{+}}\right) & \left(+N_{\mathrm{Arg}})\&(+N_{{\mathrm{NH}}_{4}^{+}}\right) & \left(+N_{\mathrm{Glu}})\&(+N_{{\mathrm{NH}}_{4}^{+}}\right) \end{array}\right]$$

The rows (*i*) and columns (*j*) of Matrix (1) are denoted by *r_i_* and *c_j_* (*i*, *j* = 1, 2, 3), respectively. The ‘&’ indicated the combination of two different N sources. For example, *a*_11_ refers to the treatment without organic N, ‘−N_O_’ and inorganic N (KNO_3_/NH_4_Cl) ‘−N_I_’; *a*_12_ refers to the treatment with organic N (Arg) ‘+N_Arg_’, but without inorganic N (KNO_3_/NH_4_Cl) ‘−N_I_’; *a*_13_ refers to the treatment with organic N (Glu) ‘+N_Glu_’, but without inorganic N (KNO_3_/NH_4_Cl) ‘−N_I_’.

To compare the contribution of organic and inorganic N to Pro content, Matrix (1) was performed with the elementary row (*r*) transformation, i.e., *r*_2_ − *r*_1_, *r*_3_ − *r*_1_, and *r*_3_ − *r*_2_. Therefore, the following three matrices were obtained:

(2) $$\left[\begin{array}{ccc} \left(+N_{{\mathrm{NO}}_{3}^{-}})-(-N_{I}\right) & \left(+N_{{\mathrm{NO}}_{3}^{-}})-(-N_{I}\right) & \left(+N_{{\mathrm{NO}}_{3}^{-}})-(-N_{I}\right)\\ \left(-N_{O})+(+N_{{\mathrm{NO}}_{3}^{-}}\right) & \left(+N_{\mathrm{Arg}})+(+N_{{\mathrm{NO}}_{3}^{-}}\right) & \left(+N_{\mathrm{Arg}})+(+N_{{\mathrm{NO}}_{3}^{-}}\right)\\ \left(-N_{O})+(+N_{{\mathrm{NH}}_{4}^{+}}\right) & \left(+N_{\mathrm{Arg}})+(+N_{{\mathrm{NH}}_{4}^{+}}\right) & \left(+N_{\mathrm{Arg}})+(+N_{{\mathrm{NH}}_{4}^{+}}\right) \end{array}\right]$$

(3) $$\left[\begin{array}{ccc} \left(+N_{{\mathrm{NH}}_{4}^{+}})-(-N_{I}\right) & \left(+N_{{\mathrm{NH}}_{4}^{+}})-(-N_{I}\right) & \left(+N_{{\mathrm{NH}}_{4}^{+}})-(-N_{I}\right)\\ \left(-N_{O})+(+N_{{\mathrm{NO}}_{3}^{-}}\right) & \left(+N_{\mathrm{Arg}})+(+N_{{\mathrm{NO}}_{3}^{-}}\right) & \left(+N_{\mathrm{Glu}})+(+N_{{\mathrm{NO}}_{3}^{-}}\right)\\ \left(-N_{O})+(+N_{{\mathrm{NH}}_{4}^{+}}\right) & \left(+N_{\mathrm{Arg}})+(+N_{{\mathrm{NH}}_{4}^{+}}\right) & \left(+N_{\mathrm{Glu}})+(+N_{{\mathrm{NH}}_{4}^{+}}\right) \end{array}\right]$$

(4) $$\left[\begin{array}{ccc} \left(-N_{O})+(-N_{I}\right) & \left(+N_{\mathrm{Arg}})+(-N_{I}\right) & \left(+N_{\mathrm{Glu}})+(-N_{I}\right)\\ \left(+N_{{\mathrm{NH}}_{4}^{+}})-(+N_{{\mathrm{NO}}_{3}^{-}}\right) & \left(+N_{{\mathrm{NH}}_{4}^{+}})-(+N_{{\mathrm{NO}}_{3}^{-}}\right) & \left(+N_{{\mathrm{NH}}_{4}^{+}})-(+N_{{\mathrm{NO}}_{3}^{-}}\right)\\ \left(-N_{O})+(+N_{{\mathrm{NH}}_{4}^{+}}\right) & \left(+N_{\mathrm{Arg}})+(+N_{{\mathrm{NH}}_{4}^{+}}\right) & \left(+N_{\mathrm{Glu}})+(+N_{{\mathrm{NH}}_{4}^{+}}\right) \end{array}\right]$$

*r*_1_ in Matrix (2), *r*_1_ in Matrix (3) and *r*_2_ in Matrix (4) were all extracted to obtain Matrix (5):

(5) $$\left[\begin{array}{ccc} \left(+N_{{\mathrm{NO}}_{3}^{-}})-(-N_{I}\right) & \left(+N_{{\mathrm{NO}}_{3}^{-}})-(-N_{I}\right) & \left(+N_{{\mathrm{NO}}_{3}^{-}})-(-N_{I}\right)\\ \left(+N_{{\mathrm{NH}}_{4}^{+}})-(-N_{I}\right) & \left(+N_{{\mathrm{NH}}_{4}^{+}})-(-N_{I}\right) & \left(+N_{{\mathrm{NH}}_{4}^{+}})-(-N_{I}\right)\\ \left(+N_{{\mathrm{NH}}_{4}^{+}})-(+N_{{\mathrm{NO}}_{3}^{-}}\right) & \left(+N_{{\mathrm{NH}}_{4}^{+}})-(+N_{{\mathrm{NO}}_{3}^{-}}\right) & \left(+N_{{\mathrm{NH}}_{4}^{+}})-(+N_{{\mathrm{NO}}_{3}^{-}}\right) \end{array}\right]$$

where *r*_1_ is the contribution of inorganic N (KNO_3_) ‘+N_NO3_^−^’ to Pro content; *r*_2_ is the contribution of inorganic N (NH_4_Cl) ‘+N_NH4_^+^’ to Pro content; *r*_3_ is the difference between the contribution of inorganic N (NH_4_Cl) ‘+N_NH4_^+^’ and inorganic N (KNO_3_) ‘+N_NO3_^−^’ to Pro content.

Additionally, Matrix (1) was performed with the elementary column (*c*) transformations, i.e., *c*_2_ − *c*_1_, *c*_3_ − *c*_1_, and *c*_3_ − *c*_2_. Therefore, the following three matrices were obtained:

(6) $$\left[\begin{array}{ccc} \left(+N_{\mathrm{Arg}})-(-N_{O}\right) & \left(+N_{\mathrm{Arg}})+(-N_{I}\right) & \left(+N_{\mathrm{Glu}})+(-N_{I}\right)\\ \left(+N_{\mathrm{Arg}})-(-N_{O}\right) & \left(+N_{\mathrm{Arg}})+(+N_{{\mathrm{NO}}_{3}^{-}}\right) & \left(+N_{\mathrm{Glu}})+(+N_{{\mathrm{NO}}_{3}^{-}}\right)\\ \left(+N_{\mathrm{Arg}})-(-N_{O}\right) & \left(+N_{\mathrm{Arg}})+(+N_{{\mathrm{NH}}_{4}^{+}}\right) & \left(+N_{\mathrm{Glu}})+(+N_{{\mathrm{NH}}_{4}^{+}}\right) \end{array}\right]$$

(7) $$\left[\begin{array}{ccc} \left(+N_{\mathrm{Glu}})-(-N_{O}\right) & \left(+N_{\mathrm{Arg}})+(-N_{I}\right) & \left(+N_{\mathrm{Glu}})+(-N_{I}\right)\\ \left(+N_{\mathrm{Glu}})-(-N_{O}\right) & \left(+N_{\mathrm{Arg}})+(+N_{{\mathrm{NO}}_{3}^{-}}\right) & \left(+N_{\mathrm{Glu}})+(+N_{{\mathrm{NO}}_{3}^{-}}\right)\\ \left(+N_{\mathrm{Glu}})-(-N_{O}\right) & \left(+N_{\mathrm{Arg}})+(+N_{{\mathrm{NH}}_{4}^{+}}\right) & \left(+N_{\mathrm{Glu}})+(+N_{{\mathrm{NH}}_{4}^{+}}\right) \end{array}\right]$$

(8) $$\left[\begin{array}{ccc} \left(-N_{O})+(-N_{I}\right) & \left(+N_{\mathrm{Glu}})-(+N_{\mathrm{Arg}}\right) & \left(+N_{\mathrm{Glu}})+(-N_{I}\right)\\ \left(-N_{O})+(+N_{{\mathrm{NO}}_{3}^{-}}\right) & \left(+N_{\mathrm{Glu}})-(+N_{\mathrm{Arg}}\right) & \left(+N_{\mathrm{Glu}})+(+N_{{\mathrm{NO}}_{3}^{-}}\right)\\ \left(-N_{O})+(+N_{{\mathrm{NH}}_{4}^{+}}\right) & \left(+N_{\mathrm{Glu}})-(+N_{\mathrm{Arg}}\right) & \left(+N_{\mathrm{Glu}})+(+N_{{\mathrm{NH}}_{4}^{+}}\right) \end{array}\right]$$

*c*_1_ in Matrix (6), *c*_1_ in Matrix (7) and *c*_2_ in Matrix (8) were all extracted to obtain Matrix (9):

(9) $$\left[\begin{array}{ccc} \left(+N_{\mathrm{Arg}})-(-N_{O}\right) & \left(+N_{\mathrm{Glu}})-(-N_{O}\right) & \left(+N_{\mathrm{Glu}})-(+N_{\mathrm{Arg}}\right)\\ \left(+N_{\mathrm{Arg}})-(-N_{O}\right) & \left(+N_{\mathrm{Glu}})-(-N_{O}\right) & \left(+N_{\mathrm{Glu}})-(+N_{\mathrm{Arg}}\right)\\ \left(+N_{\mathrm{Arg}})-(-N_{O}\right) & \left(+N_{\mathrm{Glu}})-(-N_{O}\right) & \left(+N_{\mathrm{Glu}})-(+N_{\mathrm{Arg}}\right) \end{array}\right]$$

where *c*_1_ is the contribution of organic N (Arg) ‘+N_Arg_’ to Pro content; *c*_2_ is the contribution of organic N (Glu) ‘+N_Glu_’ to Pro content; *c*_3_ is the difference between the contribution of organic N (Glu) ‘+N_Glu_’ and organic N (Arg) ‘+N_Arg_’ to Pro content.

Next, ‘(−N_O_) and (−N_I_)’ was set to 0, and Matrix (9) was subtracted from Matrix (5) to yield Matrix (10):

(10) $$\left[\begin{array}{ccc} \left(+N_{\mathrm{Arg}})-(+N_{{\mathrm{NO}}_{3}^{-}}\right) & \left(+N_{\mathrm{Glu}})-(+N_{{\mathrm{NO}}_{3}^{-}}\right) & \left(+N_{\mathrm{Glu}})-(+N_{\mathrm{Arg}})-(+N_{{\mathrm{NO}}_{3}^{-}}\right)\\ \left(+N_{\mathrm{Arg}})-(+N_{{\mathrm{NH}}_{4}^{+}}\right) & \left(+N_{\mathrm{Glu}})-(+N_{{\mathrm{NH}}_{4}^{+}}\right) & \left(+N_{\mathrm{Glu}})-(+N_{\mathrm{Arg}}\mathrm{xFFFD}-(+N_{{\mathrm{NH}}_{4}^{+}}\right)\\ \left(+N_{\mathrm{Arg}})-(+N_{{\mathrm{NH}}_{4}^{+}})-(+N_{{\mathrm{NO}}_{3}^{-}}\right) & \left(+N_{\mathrm{Glu}})-(+N_{{\mathrm{NH}}_{4}^{+}})-(+N_{{\mathrm{NO}}_{3}^{-}}\right) & \left(+N_{\mathrm{Glu}})-(+N_{\mathrm{Arg}})-(+N_{{\mathrm{NH}}_{4}^{+}})-(+N_{{\mathrm{NO}}_{3}^{-}}\right) \end{array}\right]$$

Then, *a*_11_, *a*_12_, *a*_21_, and *a*_22_ from Matrix (10) were extracted to form Matrix (11):

(11) $$\left[\begin{array}{cc} \left(+N_{\mathrm{Arg}})-(+N_{{\mathrm{NO}}_{3}^{-}}\right) & \left(+N_{\mathrm{Glu}})-(+N_{{\mathrm{NO}}_{3}^{-}}\right)\\ \left(+N_{\mathrm{Arg}})-(+N_{{\mathrm{NH}}_{4}^{+}}\right) & \left(+N_{\mathrm{Glu}})-(+N_{{\mathrm{NH}}_{4}^{+}}\right) \end{array}\right]$$

where *a*_11_ represents the difference between the contribution of ‘Arg and NO_3_^−^’ to Pro content; *a*_12_ represents the difference between the contribution of ‘Glu and NO_3_^−^’ to Pro content; *a*_21_ represents the difference between the contribution of ‘Arg and NH_4_^+^’ to Pro content; *a*_22_ represents the difference between the contribution of ‘Glu and NH_4_^+^’ to Pro content. In addition, the values of *a*_3*j*_ in Matrix (5), *a_i_*_3_ in Matrix (9), and *a*_11_, *a*_12_, *a*_21_ and *a*_22_ in Matrix (11) were used to reflect the contribution of organic (Arg/Glu) and inorganic (KNO_3_/NH_4_Cl) N alone to Pro content.

## 3. Results

### 3.1. Pro Content in Rice Tissues under ‘Cr(III)+(−N_I_)’ Treatments

**Figure 2:**
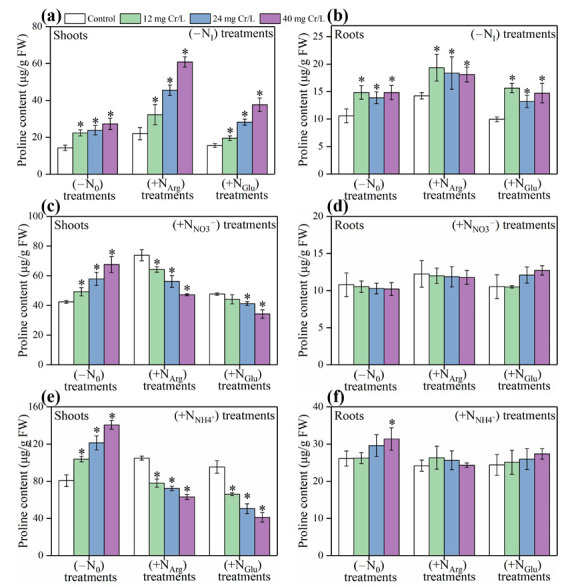
The content of Pro in shoots and roots of rice seedlings under ‘Cr(III)+(−N_I_)’, ‘Cr(III)+(+N_NO3_^−^)’, and ‘Cr(III)+(+N_NH4_^+^)’ treatments. The Pro content in (**a**) shoots and (**b**) roots of rice seedlings under ‘Cr(III)+(−N_I_)’ treatments. The Pro content in (**c**) shoots and (**d**) roots of rice seedlings under ‘Cr(III)+(+N_NO3_^−^)’ treatments. The Pro content in (**e**) shoots and (**f**) roots of rice seedlings under ‘Cr(III)+(+N_NH4_^+^)’ treatments. “*” indicate significant differences compared with the control group (*p* < 0.05).

Under ‘Cr(III)+(−N_I_)’ treatments, the Pro content in shoots of rice seedlings cultivated with ‘−N_O_’, ‘+N_Arg_’, and ‘+N_Glu_’ was determined to be “14.30 to 27.28 μg/g FW”, “21.96 to 60.85 μg/g FW”, and “15.53 to 37.65 μg/g FW”, respectively (Figure 2a). These results reveal that Cr(III) treatment increased Pro content in a dose-dependent manner compared with the untreated rice plants. The addition of arginine and glutamate significantly improved Pro content under Cr(III) treatments in rice seedlings in a dose-dependent manner.

The Pro content in roots of rice seedlings cultivated with ‘−N_O_’, ‘+N_Arg_’, and ‘+N_Glu_’ was “10.59 to 14.82 μg/g FW”, “14.23 to 19.35 μg/g FW”, and “9.95 to 15.64 μg/g FW”, respectively (Figure 2b). This shows that Pro content was lower in roots compared with shoots of rice seedlings under the same treatments. Higher proline content under Cr(III) treatments was observed in arginine-supplemented rice plants.

### 3.2. Pro Content in Rice Tissues under ‘Cr(III)+(+N_NO3_^−^)’ Treatments

Under ‘Cr(III)+(+N_NO3_^−^)’ treatments, the Pro content in shoots of rice seedlings cultivated with ‘−N_O_’, ‘+N_Arg_’, and ‘+N_Glu_’ was determined to be “42.40 to 67.55 μg/g FW”, “47.16 to 73.83 μg/g FW”, and “34.24 to 47.70 μg/g FW”, respectively (Figure 2c). Although the increase in proline content was in a dose-dependent manner in Cr(III)+(−N_O_) treatments, proline content in Cr(III)+(+N_NO3_^−^) treatments supplemented with arginine and glutamate showed an opposite trend. 

The Pro content in roots of rice seedlings cultivated with ‘−N_O_’, ‘+N_Arg_’, and ‘+N_Glu_’ was “10.21 to 10.78 μg/g FW”, “11.78 to 12.24 μg/g FW”, and “10.50 to 12.72 μg/g FW”, respectively (Figure 2d). There was no significant difference in Pro content of shoots among all the treatments.

### 3.3. Pro Content in Rice Tissues under ‘Cr(III)+(+N_NH4_^+^)’ Treatments

Under ‘Cr(III)+(+N_NH4_^+^)’ treatment, the Pro content in shoots of rice seedlings cultivated with ‘−N_O_’, and ‘+N_Arg_’, ‘+N_Glu_’ was determined to be “80.61 to 140.71 μg/g FW”, “63.11 to 104.94 μg/g FW”, and “41.23 to 95.38 μg/g FW”, respectively (Figure 2e). It was observed that increase in Cr concentration significantly increased Pro content under Cr(III)+(+N_NH4_^+^) treatments without arginine and glutamate. However, the supplementation of arginine and glutamate showed an opposite trend for Pro accumulation in rice roots under Cr(III)+(+N_NH4_^+^) treatments.

The Pro content in roots of rice seedlings cultivated with ‘−N_O_’, ‘+N_Arg_’, and ‘+N_Glu_’ was “26.12 to 31.39 μg/g FW”, “24.18 to 26.34 μg/g FW”, and “24.39 to 27.37 μg/g FW”, respectively(Figure 2f). There was no significant difference in Pro content of shoots among all the treatments.

### 3.4. The Contribution of Organic and Inorganic N Application to Pro Content in Rice Seedlings

Herein, we take the treatment with 0.0 mg Cr/L application as an example to predict the contribution of organic and inorganic N alone and in combination with Pro content in rice seedlings (Table 1).

#### 3.4.1. Pro Content in Shoots of Cr(III)-Treated Rice Seedlings

Using the measured data of Pro content in rice shoots, Matrix (12) was formulated as follows:

(12) $$\left[\begin{array}{ccc} 14.30 & 21.96 & 15.53\\ 42.40 & 73.83 & 47.70\\ 80.61 & 104.94 & 95.38 \end{array}\right]$$

As shown in Matrix (12), the contribution of the ‘N_I_ + N_O_’ application to Pro content was in the following order: ‘N_NH4_^+^ + N_Arg_’ > ‘N_NH4_^+^ + N_Glu_’ > ‘N_NO3_^−^ + N_Arg_’ > ‘N_NO3_^−^ + N_Glu_’.

To compare the contribution of inorganic N (N_NH4_^+^/N_NO3_^−^) application to Pro content, the result is shown in Matrix (13):

(13) $$\left[\begin{array}{ccc} 28.10 & 51.87 & 32.17\\ 66.31 & 82.98 & 79.85\\ 38.21 & 31.11 & 47.68 \end{array}\right]$$

According to the calculation presented in the third row (*r*_3_) of Matrix (13), the N_NH4_^+^ application showed a higher contribution to Pro content than the N_NO3_^−^ application.

Then, Matrix (14) can be obtained as follows:

(14) $$\left[\begin{array}{ccc} 7.66 & 1.23 & -6.43\\ 31.43 & 5.30 & -26.13\\ 24.33 & 14.77 & -9.56 \end{array}\right]$$

Based on the results presented in the third column (*c*_3_) of Matrix (14), the contribution of the N_Arg_ application to Pro content is higher than that of the N_Glu_ application. 

The contribution of each individual organic/inorganic N to Pro content was calculated based on Matrix (15), and the result is shown as follows:

(15) $$\left[\begin{array}{cc} -20.44 & -50.64\\ -34.88 & -77.68 \end{array}\right]$$

The contribution of inorganic/organic N application to Pro content was in the following manner: ‘N_NO3_^−^’ > ‘N_Arg_’, ‘N_NO3_^−^’ > ‘N_Glu_’, ‘N_NH4_^+^’ > ‘N_Arg_’, and ‘N_NH4_^+^’ > ‘N_Glu_’. Accordingly, based on the values of *a*_3*j*_ in Matrix (13), *a_i_*_3_ in Matrix (14), and *a*_11_, *a*_12_, *a*_21_, and *a*_22_ in Matrix (15), the contribution of inorganic/organic N application to Pro content was in the following order: ‘N_NH4_^+’^ > ‘N_NO3_^−’^ > ‘N_Arg_’ > ‘N_Glu_’.

#### 3.4.2. Pro Content in Roots of Cr(III)-Treated Rice Seedlings

Using the measured data of Pro content in rice roots, Matrix (16) was formulated as follows:

(16) $$\left[\begin{array}{ccc} 10.59 & 14.23 & 9.95\\ 10.78 & 12.24 & 10.52\\ 26.12 & 24.18 & 24.39 \end{array}\right]$$

It can be seen from Matrix (16) that the contribution of ‘N_I_ + N_O_’ application to Pro content was in the following order: ‘N_NH4_^+^ + N_Arg_’ ≈ ‘N_NH4_^+^ + N_Glu_’ > ‘N_NO3_^−^ + N_Arg_’ ≈ ‘N_NO3_^−^ + N_Glu_’.

Matrix (17) was obtained as follows:

(17) $$\left[\begin{array}{ccc} 0.19 & -1.99 & 0.57\\ 15.53 & 9.95 & 14.44\\ 15.34 & 11.94 & 13.87 \end{array}\right]$$

According to the third row of Matrix (17), the contribution of inorganic N sources to Pro content was in the following order: ‘N_NH4_^+^’ > ‘N_NO3_^−^’.

Then, Matrix (18) was obtained as follows:

(18) $$\left[\begin{array}{ccc} 3.64 & -0.64 & -4.28\\ 1.46 & -0.26 & -1.72\\ -1.94 & -1.73 & 0.21 \end{array}\right]$$

Based on the third column of Matrix (18), the contribution of organic N sources to Pro content was in the following order: ‘N_Arg_’ > ‘N_Glu_’.

Then, Matrix (19) was generated:

(19) $$\left[\begin{array}{cc} 3.45 & 1.35\\ -14.07 & -10.21 \end{array}\right]$$

Matrix (19) shows that the contribution of inorganic or organic N sources to Pro content was in the following manner: ‘N_Arg_’ > ‘N_NO3_^−^’, ‘N_Glu_’ > ‘N_NO3_^−^’, ‘N_Arg_’ > ‘N_NH4_^+^’, and ‘N_NH4_^+^’ > ‘N_Glu_’. Similarly, based on the values of *a*_3*j*_ in Matrix (17), *a_i_*_3_ in Matrix (18), and *a*_11_, *a*_12_, *a*_21_, and *a*_22_ in Matrix (19), the contribution of inorganic or organic N sources to Pro content was in the following order: ‘N_Arg_’ > ‘N_NH4_^+^’ > ‘N_Glu_’ > ‘N_NO3_^−^’. Moreover, we also calculated the contribution of inorganic and organic N sources alone and in combination with Pro content in both roots and shoots of rice seedlings under 12, 24, and 40 mg Cr/L treatments (Table 2 and Table 3). The application of N sources alone, ‘N_NH4_^+^’, showed the largest contribution to Pro content in rice shoots under different concentrations of Cr(III), followed by ‘N_NO3_^−^’, ‘N_Arg_’, and ‘N_Glu_’ application, while ‘N_Arg_’ application displayed the largest contribution to the Pro content in rice roots under different concentrations of Cr(III), followed by ‘N_NH4_^+^’, ‘N_Glu_’, and ‘N_NO3_^−^’ applications. Regarding the application of N sources in combination, ‘N_NH4_^+^ + N_Arg_’ application showed the largest contribution to Pro content in both roots and shoots of rice seedlings under different concentrations of Cr(III), while ‘N_NO3_^−^ + N_Glu_’ application contributed the least to Pro content in both roots and shoots of Cr(III)-treated rice seedlings. These results suggested that the contribution of different N sources to Pro content in Cr(III)-treated rice seedlings is different.

## 4. Discussion

The toxic effects of Cr on plants have been extensively reported, such as delaying seed germination, inhibiting root growth, reducing plant height, changing the antioxidative enzyme activities, nutrient elements uptake, and amino acids content [18,19]. In recent years, many strategies have been proposed to curtail the negative effects of Cr pollution on plants, wherein plant growth regulators (PGRs) are considered as one of the most practical and cost-effective methods [20,21]. However, great difficulties are often faced in the selection of PGRs and for evaluating their efficiency in field trials [22]. In this study, we investigated the effect of different nitrogenous compounds as substrates for synthesizing Pro in Cr(III)-treated rice seedlings using MBMM. The endogenous level of Pro in plants is highly dependent on the plant’s growth conditions [23]. Previous studies also demonstrated that the aerial part is the major site of Pro synthesis in rice plants [24,25]. Accordingly, we have also observed a higher concentration of Pro in shoots of rice seedlings (Figure 2). On the other hand, Pro concentration increases with an increase in the intensity of stress until it reaches a threshold level. Pro acts as an excellent osmolyte, a metal chelator, an antioxidative defense molecule, and a signaling molecule which reduces the effects of stress conditions [10,25]. Herein, we observed that the accumulation of Pro in shoots of rice seedlings was dependent on the doses of Cr(III) exposure (Figure 2). 

Previously, it has been reported that the content of amino acids, i.e., Pro, in plants is highly dependent on N nutrition [11]. In addition, plant growth highly depends on the forms of N present in the growth media, the amount of N available, and the plant species specifically under stress conditions [26,27]. In our case, we noticed that the response of Pro content was different between organic N-fed and inorganic N-fed seedlings under Cr(III) exposure, indicating that the contribution of these N compounds to Pro content is different [12]. Notably, under ‘Cr(III)+(−N_I_)’ treatments, relatively higher Pro content was observed in ‘+N_Arg_’-fed and ‘+N_Glu_’-fed rice seedlings than ‘−N_O_’-fed rice seedlings (Figure 2a). The possible reasons behind the higher Pro accumulation might be the supplication of arginine and glutamate amino acids, which are the precursors involved in Pro synthesis [28]. Between Arg and Glu, Arg contributed more to Pro content in rice seedlings under Cr(III) stress, which suggests that Arg is more effective than Glu in the synthesis of Pro specifically under stress conditions. This may be due to the degradation of enzymes responsible for Glu pathway of Pro synthesis under Cr stress. Thus, Arg (ornithine) pathway is considered as an alternative to the Glu pathway for Pro synthesis, which continues under stress conditions [29,30] and may have increased Pro content in Arg-treated seedlings. In addition, Arg plays an important role in the nitrogen (N) cycle because it has the highest ratio of N to carbon among amino acids [31]. Supplementation of N sources specifically ammonium increased Pro content in rice seedlings under Cr(III) stress mainly in ‘−N_O_’ treatments (Figure 2) because Cr stress affects N metabolism which ultimately reduces Pro synthesis [13]. In addition, ammonium is mostly favored over nitrate by rice plants which increases Pro over nitrate supplementation. The decreasing trend of Pro in Arg and Glu supplemented ‘Cr(III)+(+N_NO3_^−^)’ and ‘Cr(III)+(+N_NH4_^+^)’ treatments (Figure 2c,e) is complex and needs to be studied further. There could be several possible reasons for this trend; one of the possible reasons is that chromium may react with ammonium to produce precipitates of chromium hydroxide which limit N uptake and subsequently reduce Pro content. The formation of chromium hydroxide led to a further increase in Cr stress in the solution while plants were already in Cr stress [32]. Moreover, the application of NO_3_^−^ and NH_4_^+^ increased the Pro content in ‘−N_O_’-fed rice seedlings, but reduced the Pro content in ‘+N_Arg_’ and ‘+N_Glu_’-fed seedlings, suggesting that the utilization and conversion of these inorganic and organic N sources into Pro synthesis in Cr(III)-treated rice seedlings is different. The content of Pro in both roots and shoots of rice seedlings under ‘Cr(III)+(+N_NH4_^+^)’ treatments was significantly higher (‘N_NH4_^+^’ > ‘N_NO3_^−^’ > ‘N_Arg_’ > ‘N_Glu_’) than other treatments, suggesting that ‘NH_4_^+^’ is the preferred N source that contributes to Pro synthesis in rice plants under Cr(III) stress. Therefore, Cr(III) exposure did not change the N preference in the rice, because rice is an NH_4_^+^-like crop. The uptake and assimilation rate of NH_4_^+^ by rice plants is more rapid than other N sources [33,34]. Moreover, NH_4_^+^ is mainly assimilated in rice roots [6], while NO_3_^−^ is chiefly assimilated in rice shoots [35]. 

In addition, we found that the content of Pro increased in rice shoots under Cr(III) treatments with ‘+N_NO3_^−^’ or ‘+N_NH4_^+^’ application. However, the content of Pro decreased in rice shoots under “Cr(III)+(+N_NO3_^−^)” and “Cr(III)+(+N_NH4_^+^)” treatments with ‘+N_Arg_’ or ‘+N_Glu_’ application. MBMM also suggested that the relatively higher content of Pro in rice shoots treated with N source alone was higher than that of treated with N source in combination. This may be due to the (1) application of organic N sources, influencing the uptake and metabolism of inorganic N in rice seedlings; (2) application of organic N sources, promoting the catabolism of Pro in rice seedlings. That is to say, the limiting steps of Pro metabolism in plants are different under Cr(III) stress with different N sources supplied [10,36]. To maintain the homeostasis of the internal environment, plants should rapidly metabolize the Pro to maintain normal N levels against Cr(III) stress [36]. In the future, more physiological, biochemical, and molecular studies should be carried out to reveal the effects of different N sources on Pro synthesis under Cr(III) stress.

## 5. Conclusions

The present study was conducted to analyze the role of endogenous proline as a bioindicator in the regulation of Cr stress in rice plants grown in different nitrogenous conditions. The content of Pro in rice seedlings depended on the doses of Cr(III) exposure and different N sources. The application of ‘N_NH4_^+^’ had the largest contribution to the content of Pro in rice shoots under Cr(III) stress, while ‘N_Arg_’ application had the largest contribution to the content of Pro in rice roots under Cr(III) stress. The combined application of ‘N_NH4_^+^ + N_Arg_’ had the largest contribution to the content of Pro in both roots and shoots of rice seedlings under Cr(III) stress. In contrast, ‘N_NO3_^−^ + N_Glu_’ application contributed the least to the content of Pro in Cr(III)-treated rice seedlings. In conclusion, plants can regulate the content of Pro in plant tissues to cope with the potential threat induced by Cr(III) exposure under different nutritional N sources.

## Figures and Tables

**Table 1:** The ranking of the contribution of organic and inorganic N to Pro content in rice seedlings.

Treatments	Organic and Inorganic N Treatments Alone		Organic and Inorganic N Treatments in Combination	
	Shoot	Root	Shoot	Root
Control	N_NH4_^+^ > N_NO3_^−^ > N_Arg_ > N_Glu_	N_Arg_ > N_NH4_^+^ > N_Glu_ > N_NO3_^−^	N_NH4_^+^ + N_Arg_ > N_NH4_^+^ + N_Glu_ > N_NO3_^−^ + N_Arg_ > N_NO3_^−^ + N_Glu_	N_NH4_^+^ + N_Arg_ ≈ N_NH4_^+^ + N_Glu_ > N_NO3_^−^ + N_Arg_ ≈ N_NO3_^−^ + N_Glu_
12 mg Cr/L	N_NH4_^+^ > N_NO3_^−^ > N_Arg_ > N_Glu_	N_Arg_ > N_NH4_^+^ > N_Glu_ > N_NO3_^−^	N_NH4_^+^ + N_Arg_ > N_NH4_^+^ + N_Glu_ > N_NO3_^−^ + N_Arg_ > N_NO3_^−^ + N_Glu_	N_NH4_^+^ + N_Arg_ ≈ N_NH4_^+^ + N_Glu_ > N_NO3_^−^ + N_Arg_ ≈ N_NO3_^−^ + N_Glu_
24 mg Cr/L	N_NH4_^+^ > N_NO3_^−^ > N_Arg_ > N_Glu_	N_Arg_ > N_NH4_^+^ > N_Glu_ > N_NO3_^−^	N_NH4_^+^ + N_Arg_ > N_NO3_^−^ + N_Arg_ > N_NH4_^+^ + N_Glu_ > N_NO3_^−^ + N_Glu_	N_NH4_^+^ + N_Arg_ ≈ N_NH4_^+^ + N_Glu_ > N_NO3_^−^ + N_Arg_ ≈ N_NO3_^−^ + N_Glu_
40 mg Cr/L	N_NH4_^+^ > N_NO3_^−^ > N_Arg_ > N_Glu_	N_Arg_ > N_NH4_^+^ > N_Glu_ > N_NO3_^−^	N_NH4_^+^ + N_Arg_ > N_NO3_^−^ + N_Arg_ > N_NH4_^+^ + N_Glu_ > N_NO3_^−^ + N_Glu_	N_NH4_^+^ + N_Arg_ ≈ N_NH4_^+^ + N_Glu_ > N_NO3_^−^ + N_Arg_ ≈ N_NO3_^−^ + N_Glu_
In summary	N_NH4_^+^ > N_NO3_^−^ > N_Arg_ > N_Glu_	N_Arg_ > N_NH4_^+^ > N_Glu_ > N_NO3_^−^	0 and 12 mg Cr/L treatment: N_NH4_^+^ + N_Arg_ > N_NH4_^+^ + N_Glu_ > N_NO3_^−^ + N_Arg_ > N_NO3_^−^ + N_Glu_ 24 and 40 mg Cr/L treatment: N_NH4_^+^ + N_Arg_ > N_NO3_^−^ + N_Arg_ > N_NH4_^+^ + N_Glu_ > N_NO3_^−^ + N_Glu_	N_NH4_^+^ + N_Arg_ ≈ N_NH4_^+^ + N_Glu_ > N_NO3_^−^ + N_Arg_ ≈ N_NO3_^−^ + N_Glu_

**Table 2:** The contribution of inorganic and organic N alone and in combination to Pro content in shoot of rice seedlings under 12, 24, and 40 mg Cr/L treatments.

	Treatments	(12)	(13)	(14)	(15)
Shoot	Control	$\left[\begin{array}{ccc} 14.30 & 21.96 & 15.53\\ 42.40 & 73.83 & 47.70\\ 80.61 & 104.94 & 95.38 \end{array}\right]$	$\left[\begin{array}{ccc} 28.10 & 51.87 & 32.17\\ 66.31 & 82.98 & 79.85\\ 38.21 & 31.11 & 47.68 \end{array}\right]$	$\left[\begin{array}{ccc} 7.66 & 1.23 & -6.43\\ 31.43 & 5.30 & -26.13\\ 24.33 & 14.77 & -9.56 \end{array}\right]$	$\left[\begin{array}{cc} -20.44 & -50.64\\ -34.88 & -77.68 \end{array}\right]$
	12 mgCr/L	$\left[\begin{array}{ccc} 22.39 & 32.26 & 19.57\\ 49.22 & 64.22 & 44.15\\ 103.90 & 78.06 & 66.03 \end{array}\right]$	$\left[\begin{array}{ccc} 26.83 & 31.96 & 24.58\\ 81.51 & 45.80 & 46.46\\ 54.68 & 13.84 & 21.88 \end{array}\right]$	$\left[\begin{array}{ccc} 9.87 & -2.82 & -12.69\\ 15.00 & -5.07 & -20.07\\ -25.84 & -37.87 & -12.03 \end{array}\right]$	$\left[\begin{array}{cc} -16.96 & -34.78\\ -66.51 & -50.87 \end{array}\right]$
	24 mgCr/L	$\left[\begin{array}{ccc} 23.88 & 45.53 & 28.23\\ 57.87 & 56.14 & 41.24\\ 121.33 & 72.23 & 50.46 \end{array}\right]$	$\left[\begin{array}{ccc} 33.99 & 10.61 & 13.01\\ 97.45 & 26.70 & 22.23\\ 63.46 & 16.09 & 9.22 \end{array}\right]$	$\left[\begin{array}{ccc} 21.65 & 4.35 & -17.30\\ -1.73 & -16.63 & -14.90\\ -49.10 & -70.87 & -21.77 \end{array}\right]$	$\left[\begin{array}{cc} -12.34 & -6.26\\ -99.18 & -43.33 \end{array}\right]$
	40 mgCr/L	$\left[\begin{array}{ccc} 27.28 & 60.85 & 37.65\\ 67.55 & 47.16 & 34.24\\ 140.71 & 63.11 & 41.23 \end{array}\right]$	$\left[\begin{array}{ccc} 40.27 & -13.69 & -3.41\\ 113.43 & 2.23 & 3.58\\ 73.16 & 15.95 & 6.99 \end{array}\right]$	$\left[\begin{array}{ccc} 33.57 & 10.37 & -23.20\\ -20.39 & -33.31 & -12.92\\ -77.60 & -99.48 & -21.88 \end{array}\right]$	$\left[\begin{array}{cc} -6.70 & 24.06\\ -133.82 & -35.57 \end{array}\right]$

**Table 3:** The contribution of inorganic and organic N alone and in combination to Pro content in root of rice seedlings under 12, 24, and 40 mg Cr/L treatments.

	Treatments	(16)	(17)	(18)	(19)
Root	Control	$\left[\begin{array}{ccc} 10.59 & 14.23 & 9.95\\ 10.78 & 12.24 & 10.52\\ 26.12 & 24.18 & 24.39 \end{array}\right]$	$\left[\begin{array}{ccc} 0.19 & -1.99 & 0.57\\ 15.53 & 9.95 & 14.44\\ 15.34 & 11.94 & 13.87 \end{array}\right]$	$\left[\begin{array}{ccc} 3.64 & -0.64 & -4.28\\ 1.46 & -0.26 & -1.72\\ -1.94 & -1.73 & 0.21 \end{array}\right]$	$\left[\begin{array}{cc} 3.45 & 1.35\\ -14.07 & -10.21 \end{array}\right]$
	12 mg Cr/L	$\left[\begin{array}{ccc} 14.84 & 19.35 & 15.64\\ 10.53 & 12.00 & 10.50\\ 26.24 & 26.34 & 25.10 \end{array}\right]$	$\left[\begin{array}{ccc} -4.31 & -7.35 & -5.14\\ 11.40 & 6.99 & 9.46\\ 15.71 & 14.34 & 14.60 \end{array}\right]$	$\left[\begin{array}{ccc} 4.51 & 0.80 & -3.71\\ 1.47 & -0.03 & -1.50\\ 0.10 & -1.14 & -1.24 \end{array}\right]$	$\left[\begin{array}{cc} 8.82 & 8.15\\ -9.93 & -7.02 \end{array}\right]$
	24 mg Cr/L	$\left[\begin{array}{ccc} 13.88 & 18.37 & 13.19\\ 10.28 & 11.86 & 12.09\\ 29.60 & 25.64 & 25.97 \end{array}\right]$	$\left[\begin{array}{ccc} -3.60 & -6.51 & -1.10\\ 15.72 & 7.27 & 12.78\\ 19.32 & 13.78 & 13.88 \end{array}\right]$	$\left[\begin{array}{ccc} 4.49 & -0.69 & -5.18\\ 1.58 & 1.81 & 0.23\\ -3.96 & -3.63 & 0.33 \end{array}\right]$	$\left[\begin{array}{cc} 8.09 & 5.82\\ -14.14 & -5.46 \end{array}\right]$
	40 mg Cr/L	$\left[\begin{array}{ccc} 14.82 & 18.10 & 14.71\\ 10.21 & 11.78 & 12.72\\ 31.39 & 24.34 & 27.37 \end{array}\right]$	$\left[\begin{array}{ccc} -4.61 & -6.32 & -1.99\\ 16.57 & 6.24 & 12.66\\ 21.18 & 12.56 & 14.65 \end{array}\right]$	$\left[\begin{array}{ccc} 3.28 & -0.11 & -3.39\\ 1.57 & 2.51 & 0.94\\ -7.05 & -4.02 & 3.03 \end{array}\right]$	$\left[\begin{array}{cc} 7.89 & 6.21\\ -15.00 & -3.73 \end{array}\right]$

## Data Availability

The authors acknowledge that the data presented in this study must be deposited and made publicly available in an acceptable repository before publication.

## Supplementary Materials

The following supporting information can be downloaded at: https://www.mdpi.com/article/10.3390/toxics11100803/s1, Figure S1. The standard curve between the absorbance at 520 nm and L-proline content. Series content of L-proline (0, 2, 4, 6, 8, and 10 μg/mL).
