# Supplementary material for: Mathematical Estimation of Endogenous Proline as a Bioindicator to Regulate the Stress of Trivalent Chromium on Rice Plants Grown in Different Nitrogenous Conditions
Source: Toxics. 2023 Sep 22;11(10):803. doi: 10.3390/toxics11100803 (PMC10611392; doi:10.3390/toxics11100803)
Supplement: Supplementary file 1 [file toxics-11-00803-s001.zip › toxics-2606267-supplementary.pdf]

toxics MDPI

Supplementary Materials: Mathematical estimation of endogenous proline as a bioindicator to regulate the stress of trivalent chromium on rice plants grown in different nitrogenous conditions

Chengzhi Li, Yuxi Feng, Peng Tian and Xiaozhang Yu

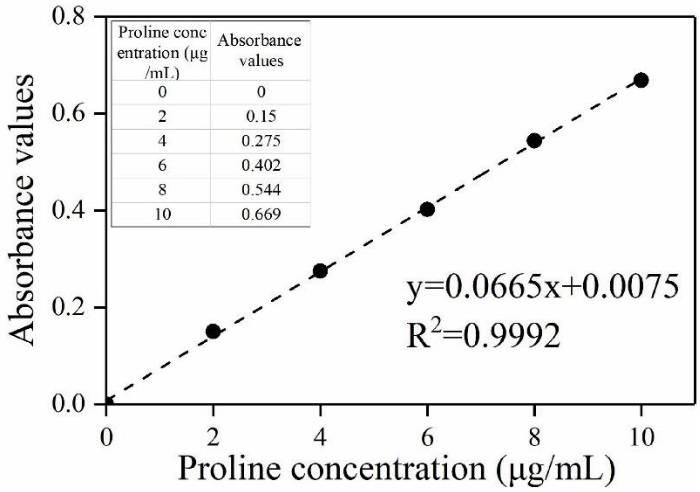

Figure S1. The standard curve between the absorbance at 520 nm and L-proline content. Series content of L-proline (0, 2, 4, 6, 8, and 10 μg/mL).
